# Long-term follow-up of scar quality and satisfaction after surgical closure of congenital abdominal wall defects: a single center perspective

**DOI:** 10.1007/s00383-025-06287-1

**Published:** 2025-12-29

**Authors:** Natalie Reindl, Sigurd Seitz, Maria Schleier, Manuel Besendörfer, Sonja Diez

**Affiliations:** 1 Department of Pediatric Surgery, Friedrich-Alexander-University of Erlangen-Nürnberg (FAU), University Hospital Erlangen, Loschgestraße 15, 91054 Erlangen, Germany; 2 Section of Pediatric Urology , Friedrich-Alexander-University of Erlangen-Nürnberg (FAU), Urology, University Hospital Erlangen, Loschgestraße 15, 91054 Erlangen, Germany; 3 Department of Pediatrics and Adolescent Medicine, Friedrich-Alexander-University of Erlangen-Nürnberg (FAU), University Hospital Erlangen, Loschgestraße 15, 91054 Erlangen, Germany

**Keywords:** Scar, Appearance, Omphalocele, Gastroschisis, Abdominal wall defect

## Abstract

**Purpose:**

Postoperative scarring remains a major concern for patients and parents following repair of congenital abdominal wall defects. This study evaluated perceptions of postoperative scars in children after omphalocele or gastroschisis repair, comparing self-assessments with parental evaluations. We hypothesized that parents perceive scarring more negatively than their children.

**Methods:**

A single-center study was conducted among patients treated for omphalocele or gastroschisis at the Department of Pediatric Surgery, University Hospital Erlangen (2001–2011). Between July 2022 and March 2023, patients and their parents completed the Patient and Observer Scar Assessment Scale (*POSAS*) 2.0, Patient Scar Assessment Questionnaire (*PSAQ*), and Short Form-36 (*SF-36*). Exclusion criteria were death or psychomotor impairment precluding reliable self-assessment.

**Results:**

Twenty-eight participants (61% gastroschisis, 39% omphalocele) were included. Parents rated scar appearance more negatively than their children (*POSAS* overall impression *p* = 0.040; appearance *p* = 0.002 in omphalocele). *PSAQ* revealed discrepancies regarding scar-related symptoms (*p* = 0.006) and satisfaction (*p* = 0.009 total; *p* = 0.038 omphalocele). Parents tended to underestimate children’s physical complaints (*p* = 0.099). No differences were found between defect types. *SF-36* indicated high overall quality of life (mean 79.9 ± 13.3).

**Conclusion:**

Significant parent–child differences exist in scar perception. Parents emphasize cosmetic concerns, whereas children report physical symptoms. Larger studies are warranted to guide tailored postoperative support.

## Introduction

Postoperative scars, visible signs of malformation, or loss of the navel can be a relevant problem for pediatric and adolescent patients. This is particularly relevant in the context of congenital abdominal wall defects, such as gastroschisis and omphalocele, which pose complex challenges for patients, parents, obstetricians, and pediatricians alike. Despite differences in pathogenesis and clinical presentation, both conditions may impair the function of multiple organ systems and typically result in scarring and potential deformity of the umbilicus after surgical closure.

Parents often worry that their children might be dissatisfied with the cosmetic outcome, particularly the appearance of the scar and navel. To address these concerns, the following study was conducted to evaluate patient satisfaction with the postoperative appearance and its impact on body image and quality of life.

Gastroschisis is an isolated abdominal wall defect with a prevalence of approximately 4 per 10,000 live births, showing a rising trend over the past three decades [1–4]. The highest prevalence is found among children of mothers under the age of 20 [1, 5].

In contrast, the prevalence of omphalocele is slightly lower, at 2 to 2.5 per 10,000 live births, with no significant change over time [3, 6, 7]. However, omphalocele is associated with an increased risk in older mothers [7].

The severity of gastroschisis is primarily determined by the presence and extent of intestinal dysfunction, most commonly caused by intestinal atresia [8–10].

Depending on the size of the abdominal wall defect, omphaloceles are classified as minor or major, with a threshold of 5 cm. A giant omphalocele is typically defined by herniation of more than 50% of the liver [11, 12]. Unlike gastroschisis, omphalocele is associated with a higher risk of additional malformations, affecting approximately 40 to 80% of caes [3, 4, 13, 14].

In cases of gastroschisis, primary closure is usually achieved in about 75% [15–17]. Among the various techniques for staged closure, the spring-loaded silo bag is the most commonly applied method [18, 19].

The present study investigates patient satisfaction following surgical closure of congenital abdominal wall defects, with focus on the subjective perception of scar appearance and the umbilicus, as well as self-reported quality of life. Additionally, parents were asked to evaluate their child’s scar to enable direct comparison. The study explores whether parental concerns about cosmetic outcomes correspond with the patient’s own perspective. It is hypothesized that parents may assess scar aesthetics more critically than the patients themselves.

## Materials and methods

### Study design

A single center survey of all neonates with the diagnosis of omphalocele and gastroschisis was conducted. Only patients with surgical abdominal wall closure performed at our center were included. Patients presenting due to secondary complaints after repair in a different hospital were excluded. To ensure average age for self-assessment (secondary school onwards), abdominal wall closure must have been performed between 01/01/2001 to 1/31/2011 for inclusion, with patient age at assessment ranging from 11 to 22 years. Exclusion criteria for our cohort were patient death and psychomotor impairments preventing self-assessment.

The study protocol was approved by the local ethics committee (Friedrich-Alexander-Universität, Erlangen-Nürnberg (FAU), No. 22–29-B) and complied with the Declaration of Helsinki. Written informed consent was obtained from each subject and next-of-kin prior to collection of data on quality of life.

A survey of both patients and their parents was conducted using the following questionnaires, which were sent to the families by post:

- *Patient and Observer Scar Assessment Scale* (POSAS) *2.0 questionnaire* [20, 21]: The patient version of the POSAS, a validated patient-reported outcome measure, was used to assess scar condition from both patients’ and parents’ perspectives. It was originated in Dutch, but is now validated for a multitude of different languages, including German. Each language version underwent psychometric validation to ensure measurement equivalence, confirming that patients understand and interpret the items similarly across cultures. The instrument captures visual, tactile, and sensory abnormalities using a 10-point numerical rating scale. Lower scores indicate a more favorable assessment of the scar. The final item evaluates the o*verall impression* of the scar, with 1 indicating similarity to normal skin and 10 indicating a marked difference. Items are combined into two sub-scores: *Pain and itching* (range 2–20) and *appearance*, which includes color, stiffness, thickness, and surface (range 4–40). These are summed to form a total score, excluding the *overall impression* item.
- *Patient Scar Assessment Questionnaire* (PSAQ) [22]: This questionnaire, administered to both patients and parents, is a validated tool for assessing patients’ perceptions of their scars across five subscales: *Appearance*,* symptoms*,* consciousness*,* satisfaction with appearance* and *satisfaction with symptoms*. Each item is scored from 1 to 4, with 1 representing the most favorable response and 4 the least favorable. Scores within each subscale are summed, so lower totals indicate higher satisfaction. At the end of each subscale, a general *overall* assessment question is included, which is not part of the summed score but serves as a clinically meaningful descriptor and allows for assessment of internal validity [23]. For these *overall* assessment items, the maximum score varies by category: *Appearance* and s*ymptoms* allow up to 5 points as the worst score, while *consciousness*,* satisfaction with appearance* and *satisfaction with symptoms* have a maximum of 4 points. The original validation was in English. Subsequent studies have translated and validated the PSAQ into other languages (e.g., Turkish, Chinese, German), following forward–backward translation and cross-cultural adaptation guidelines (per ISPOR or COSMIN standards).
- *Short form 36* (SF-36) [24]: The SF-36 is a standardized instrument comprising 36 items designed to assess self-reported health-related quality of life. It encompasses the following eight subscales, which were completed by the patient: *Physical functioning*,* role physical*,* bodily pain*,* general health*,* vitality*,* social functioning*,* role emotional* and *mental health*. The questionnaire is scored using a scoring key, with each item receiving between 0 and 100 points. A score of 100 indicates complete satisfaction, while 0 represents the poorest possible rating.

### Data management and statistical analysis

Medical history of patients was collected from patients’ documentation files. After evaluation of assessment forms as described above, overall and subcategory results were compared regarding differences between omphalocele and gastroschisis patients as well as patients and their parents. Moreover, age adjusted analysis was conducted based on a classification of adolescents (aged 11-15y) and young adults (aged 16-22y). All statistical calculations were performed using SPSS software (IBM SPSS Statistics 29.0, Armonk, New York, USA). Variables were compared using two independent sample t-tests for continuous, normally distributed data, and Mann-Whitney-U-tests for continuous, non-normally distributed data. Categorical variables were analysed using chi-square or Fisher’s exact tests, as appropriate. Results were considered statistically significant at *p* < 0.05. All results are presented with 95% confidence intervals (95% CI).

## Results

### Patient demographics and baseline characteristics

**Fig. 1:**
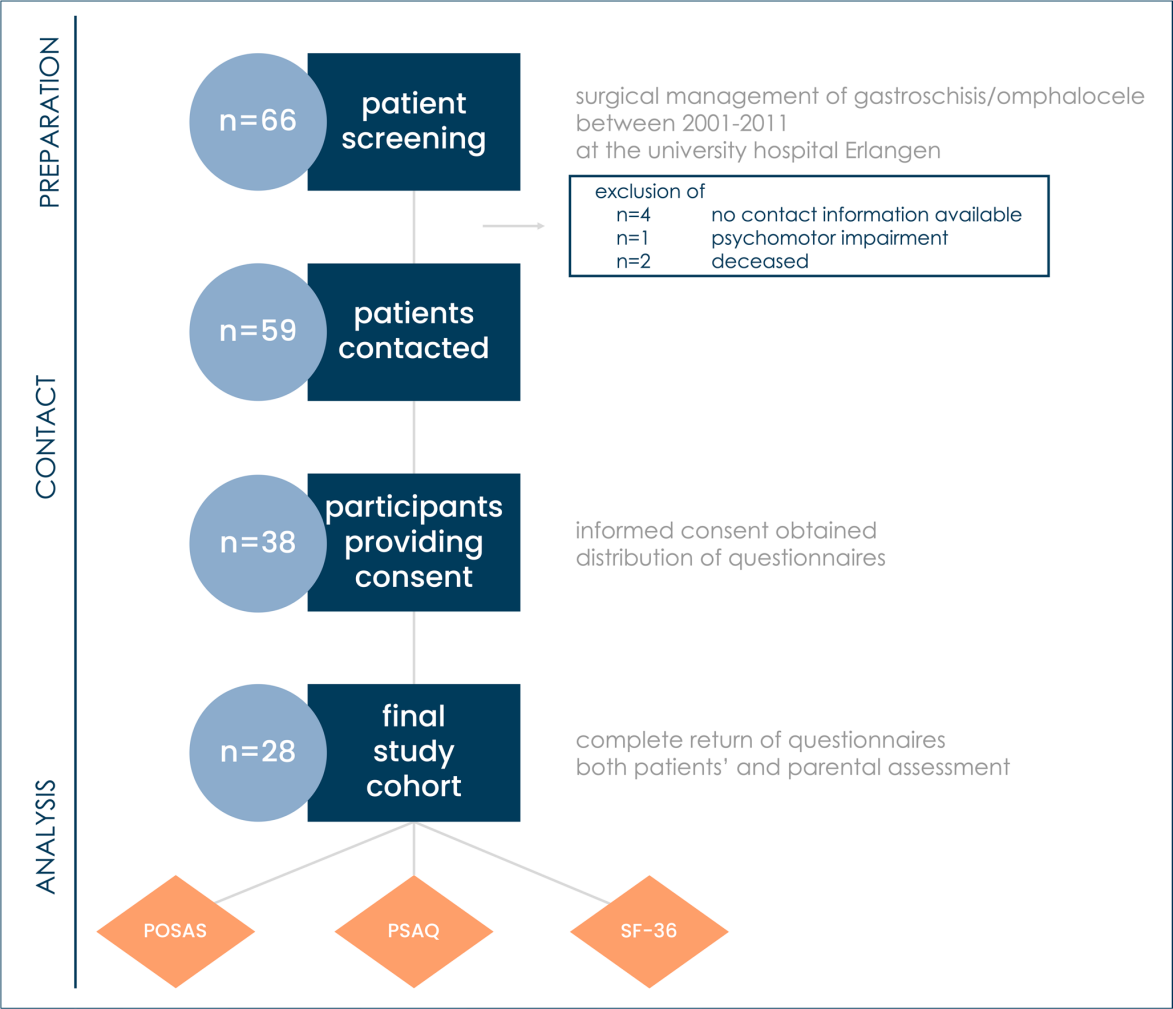
Study flow diagram. Illustration of patient recruitment leading to final analysis of 28 participants who returned all questionnaires, enabling both patient and parental assessment using the Patient and Observer Scar Assessment Scale (*POSAS*), the Patient Scar Assessment Questionnaire (*PSAQ*), and the Short Form-36 Health Survey (*SF-36*)

A total of 28 former patients meeting the inclusion criteria were included in the analysis. Exclusion of patients and determination of population based on eligibility criteria is summarized in Fig. 1. The collection of data was performed between July 2022 and March 2023, with patients being 11 to 22 years of age (median 15.5 years).

The 28 patients included in the study showed an almost equal gender distribution, with a slight predominance of females (f = 15; m = 13). Among the study cohort, 17 patients (61%) were diagnosed with gastroschisis and 11 (39%) with omphalocele. Age at time of query was comparable between the two study groups (median of 15 years in patients with gastroschisis and 16 years in patients with omphalocele, *p* = 0.732). Minor omphaloceles did not occur within the cohort and hepatic involvement, as a potential influencing factor on further scars, was balanced in group of omphalocele (55% with hepatic involvement). Associated malformations were present in 39% of cases (*n* = 11), and liver involvement was observed in 25% (*n* = 7). Primary closure during the initial surgery was achieved in 79% (*n* = 22) of patients. Abdominal wall closure without the use of prosthetic material was possible in 71% (*n* = 20). In one case, the material used could not be verified, while the remaining 25% (*n* = 7) required patch implantation. Surgical technique was comparable in all patients, as the surgical team was constant within the study’s time period. Moreover, adjusted analysis regarding surgical interventions after abdominal wall closure in gastroschisis patients (with *n* = 7 patients with following abdominal surgery and *n* = 10 patients without any further surgery) failed to reach significance.

**Fig. 2:**
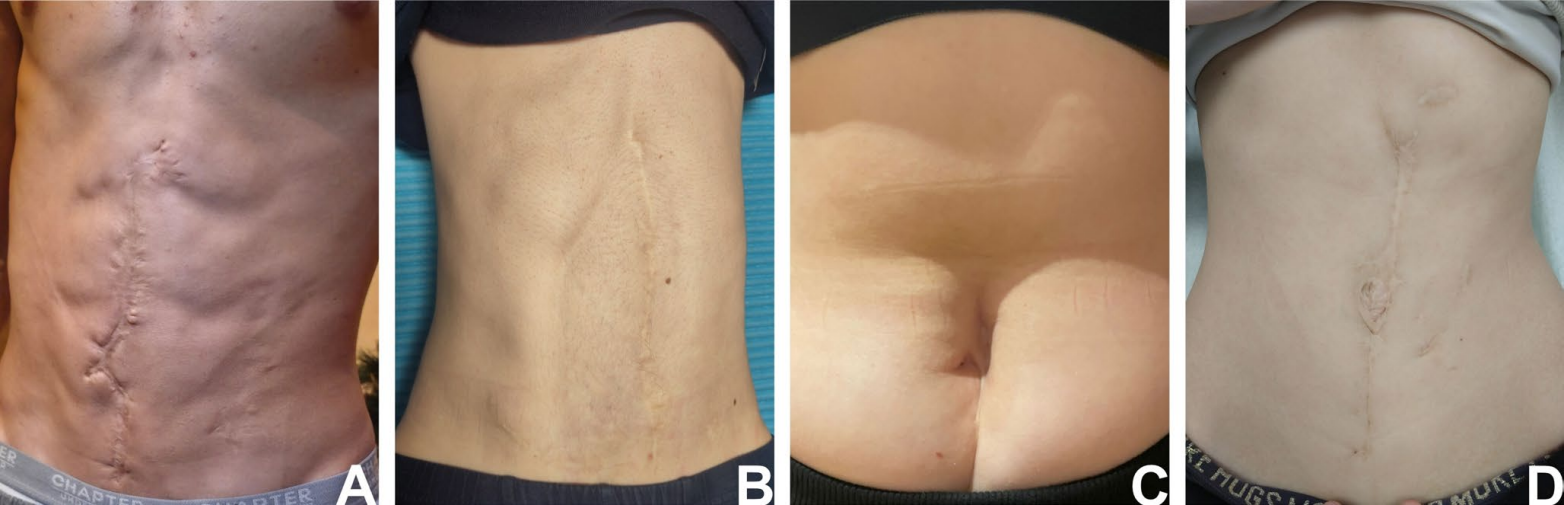
Examples of postoperative outcome within the study population. **A** 17-year-old male with gastroschisis; **B** 14-year-old male with omphalocele; **C** 15-year-old female with omphalocele; **D** 16-year-old female with omphalocele. All four patients consented to publish these representative pictures

Demographic characteristics of the study population and group-specific differences between gastroschisis and omphalocele are summarized in Table 1. Exemplary scarring is illustrated by Fig. 2.

**Table 1:** Demographic characteristics in comparison of study groups. Bold values indicate statistical significance (*p* < 0.05); underlined values indicate borderline significance (0.05 ≤ *p* < 0.10)

	Total (*n*=28)	Patients with gastroschisis (*n*=17)	Patients with omphalocele (*n*=11)	*p*-values
*Age* [median (range)]	15.5 (11–22)	15 (11–21)	16 (11–22)	0.732
Gender [n (%)]				0.246
Male	13 (46%)	6 (35%)	7 (64%)	
Female	15 (54%)	11 (65%)	4 (36%)	
*Associated Malformations [n (%)]*				*0.053*
Yes	11 (39%)	4 (24%)	7 (64%)	
No	17 (61%)	13 (76%)	4 (37%)	
*Herniation of more than one organ [n (%)]*				***0.018***
Yes	14 (50%)	12 (70%)	2 (18%)	
No	11 (39%)	4 (24%)	7 (64%)	
Unknown	3 (11%)	1 (6%)	2 (18%)	
*Liver involvement [n (%)]*				***0.007***
Yes	7 (25%)	1 (6%)	6 (55%)	
No	18 (64%)	15 (88%)	3 (27%)	
Unknown	3 (11%)	1 (6%)	2 (18%)	
*Intestinal atresia [n (%)]*				1.000
Yes	3 (11%)	2 (12%)	1 (9%)	
No	24 (86%)	14 (82%)	10 (91%)	
Unknown	1 (3%)	1 (6%)	0	
Maternal age at delivery [Median in years (range)]	27 (17–37)	22 (17–37)	29 (23–34)	***0.007***
Gestational age at delivery [Median in weeks+days (range)]	37+3 (33+0 - 39+5)	36+5 (33+0 - 38+3)	38+3 (35+2 - 39+5)	***0.005***

### POSAS

Significant differences emerged between self- and parental assessments using *POSAS*. Parents rated the *overall impression* of the scar more negatively than their children, irrespective of the underlying diagnosis (*p* = 0.040, *n* = 25). In contrast, *pain and itching* represented the only domain in which parental ratings showed a trend towards underrating compared to the children’s self-reports (*p* = 0.099, *n* = 25). A detailed overview of *POSAS* results is provided in Table 2. In the subgroup of patients with omphalocele, the difference of self- and parental assessment was even more highlighted in *appearance* (patient’s perspective (mean value of 12.00 with 95% CI of 8.10–15.90) versus parental perspective (mean value of 14.67 with 95% CI of 11.12–18.22), *p* = 0.002) and o*verall impression* (patient’s perspective (mean value of 3.33 with 95% CI of 1.37–5.29) versus parental perspective (mean value of 5.56 with 95% CI of 3.59–7.53), *p* = 0.030).

**Table 2:** POSAS results in comparison of patient’s and parental perspective. Bold values indicate statistical significance (*p* < 0.05); underlined values indicate borderline significance (0.05 ≤ *p* < 0.10)

	Patient’s perspective (*n*=25)	Parental perspective (*n*=25)	*p*-values
Symptoms [mean value (CI)]	3.16 (2.24–4.08)	2.32 (1.86–2.78)	*0.099*
Appearance [mean value (CI)]	13.56 (10.55–16.57)	16.24 (12.70–19.70)	*0.097*
Overall impression [mean value (CI)]	3.84 (2.88–4.80)	4.72 (3.80–5.64)	***0.040***
Total score [mean value (CI)]	16.72 (13.42–20.02)	18.56 (15.11–22.01)	0.178

Further analyses (comparison between study groups omphalocele versus gastroschisis/comparison between patients with gastroschisis and their parents) revealed no significant associations across all investigated parameters.

### PSAQ

*PSAQ* also showed significant differences between children and their parents regarding scar-related *symptoms* (*p* = 0.006) and *overall satisfaction with symptoms* (*p* = 0.009) in the total study population (*n* = 27). Children rated scar-related symptoms as more bothersome than their parents. This finding was confirmed in the omphalocele subgroup for *overall satisfaction with symptoms* (patient’s perspective (mean value of 2.00 with 95% CI of 1.48–2.52) versus parental perspective (mean value of 1.64 with 95% CI of 1.02–2.26, *p* = 0.038).

Further analyses comparing children and parents did not reach significance. However, borderline significance was observed for *symptoms* in both subgroups: gastroschisis (patient’s perspective (mean value of 7.94 with 95% CI of 7.13–8.75) versus parental perspective (mean value of 7.25 with 95% CI of 6.23–8.27, *p* = 0.052) and omphalocele (patient’s perspective (mean value of 8.64 with 95% CI of 7.29–9.99) versus parental perspective (mean value of 7.45 with 95% CI of 6.58–8.32, *p* = 0.058). No significant differences were found between the omphalocele and gastroschisis groups across any of the PSAQ scores. Table 3 summarizes the results of *PSAQ* analysis in the whole study group.

**Table 3:** PSAQ results in comparison of patient’s and parental perspective. Bold values indicate statistical significance (*p* < 0.05); underlined values indicate borderline significance (0.05 ≤ *p* < 0.10)

	Patient’s perspective (*n*=27)	Parental perspective (*n*=27)	*p*-values
*Appearance* [mean value (CI)]	17.85 (16.65–19.05)	18.0 (17.01–18.99)	0.733
*Appearance overall* [mean value (CI)]	2.56 (2.16–2.96)	2.3 (1.99–2.61)	0.170
*Symptoms* [mean value (CI)]	8.22 (7.53–8.91)	7.37 (6.72–8.02)	***0.006***
*Symptoms overall* [mean value (CI)]	1.52 (1.24–1.80)	1.56 (1.33–1.79)	0.663
*Consciousness* [mean value (CI)]	12.74 (11.39–14.09)	12.67 (11.43–13.91)	0.743
*Consciousness overall* [mean value (CI)]	1.56 (1.26–1.86)	1.56 (1.33–1.79)	1.000
*Satisfaction with appearance* [mean value (CI)]	14.44 (12.90–15.98)	13.63 (11.94–15.32)	0.418
*Satisfaction with appearance overall* [mean value (CI)]	2.19 (1.79–2.59)	2.04 (1.76–2.32)	0.678
*Satisfaction with symptoms* [mean value (CI)]	7.41 (6.58–8.24)	6.81 (6.10–7.52)	*0.053*
*Satisfaction with symptoms overall* [mean value (CI)]	1.74 (1.48–2.00)	1.44 (1.16–1.72)	***0.009***

### SF-36

*SF-36* showed high overall quality of life with no significant difference between gastroschisis and omphalocele with a median total score of 80.36 and 79.36, respectively (*p* = 0.864). Regarding the eight subcategories, there was a similar distribution with the lowest score being vitality (58.08) in the whole population. Results are detailed in Table 4. Moreover, analysis revealed significantly increased assessment of younger age group (patients between 11 and 15 years of age) compared to young adults (patients between 16 and 22 years of age) regarding categories of role physical (mean score at younger age 100.0 vs. mean score at older age 86.5, *p* = 0.040), general health (mean score at younger age 83.7 vs. mean score at older age 71.9, *p* = 0.044), vitality (mean score at younger age 67.0 vs. mean score at older age 29.2, *p* = 0.003), social functioning (mean score at younger age 95.1 vs. mean score at older age 75.2, *p* = 0.006), and total score (mean score at younger age 85.7 vs. mean score at older age 73.4, *p* = 0.013). Other categories of SF-36 or further questionnaires regarding age analysis failed to reach statistical significance.

**Table 4:** Results of SF-36 in comparison of study group Gastroschisis and omphalocele

	Total population (*n*=28)	Patients with gastroschisis (*n*=17)	Patients with omphalocele (*n*=11)	*p*-value
Physical functioning [mean value (CI)]	91.6 (83.8–99.4)	94.7 (86.8–102.6)	86.8 (69.5–104.1)	0.375
Role physical [mean value (CI)]	93.8 (87.3–100.2)	94.1 (84.4–103.9)	93.2 (82.4–104.0)	0.890
Bodily pain [mean value (CI)]	89.3 (82.4–96.2)	92.5 (84.8–100.2)	84.3 (69.5–99.2)	0.301
General health [mean value (CI)]	78.2 (71.6–84.8)	77.4 (67.8–86.9)	79.5 (69.0–90.1)	0.753
Vitality [mean value (CI)]	58.1 (52.1–65.4)	56.5 (46.2–66.7)	62.3 (51.6–72.9)	0.392
Social functioning [mean value (CI)]	85.7 (77.7–93.8)	84.6 (74.4–94.7)	87.5 (70.8–104.2)	0.757
Role emotional [mean value (CI)]	84.5 (63.4–126,0)	80.4 (61.6–99.2)	90.9 (70.7–111.2)	0.410
Mental health [mean value (CI)]	71.1 (44.8–97.5)	71.8 (64.4–79.2)	70.2 (59.9–80.5)	0.788
Total score [mean value (CI)]	79.9 (74.9–84.9)	80.3 (73.0–87.5)	79.3 (68.3–90.3)	0.864

## Discussion

This study provides a single-center perspective on patient and parental satisfaction and quality of life after surgical closure of congenital abdominal wall defects. To date, few studies have investigated these outcomes on a scale comparable to our study, particularly regarding direct comparisons between patient and parental perspectives. While some studies have assessed these measures beyond the first year of life, they did not differentiate between children’s and parents’ assessments [16, 25]. Our findings therefore provide novel evidence by highlighting these discrepancies and underscore the importance of including both perspectives when evaluating long-term outcomes after abdominal wall defect repair.

In the present study, we aimed to build on previous work by applying a standardized and thorough method to evaluating cosmetic outcomes after abdominal wall defect repair.

While earlier studies offered valuable insights into postoperative satisfaction, our approach extends these findings by employing validated questionnaires (*POSAS* and *PSAQ*) and systematically assessing both patient and parental perspectives. This design enables a more comprehensive and objective evaluation of long-term outcomes and perceived scar quality.

We observed an overall high satisfaction with scars and postoperative outcome of abdominal wall defects in affected children. Likewise, a survey conducted in Erlangen 2004, including patients who underwent abdominal wall closure between 1994 and 2004, demonstrated good to excellent cosmetic satisfaction in 80% of cases [16]. Kapapa et al. assessed parental satisfaction with cosmetic outcomes of the abdominal scar and reported high rates of satisfaction, reaching 81% of gastroschisis and 91% of omphalocele cases [25]. In contrast to our study, previous research primarily focused on parental or family evaluations and did not distinguish between children’s and parents’ perspectives.

Our study addressed this gap by showing that parental estimates of satisfaction do not necessarily align with the patients’ own perceptions, underscoring the importance of directly incorporating the child’s perspective when assessing long-term outcomes and quality of life after abdominal wall defect repair.

Additionally, we observed significant differences between self- and parental assessments, confirming our hypothesis that parents tend to perceive scars more negatively than the affected children in terms of both appearance and satisfaction. This finding was supported by statistically significant differences in the *POSAS* and *PSAQ* questionnaire results.

Previous studies have reported elevated stress levels among parents of prematurely born infants or neonates with cardiac defects, with the child’s appearance and behavior being identified as the primary sources of stress [26, 27]. These findings suggest that parental assessments tend to be more negative, likely because the child’s appearance itself is perceived as a major stressor.

Furthermore, our differentiation of children’s and parents’ ratings revealed a trend of differences in the assessment of physical symptoms related to the scar. Similar discrepancies have been reported in other contexts; for example, parents of pediatric patients with chronic pain have been shown to underestimate pain-related functional interference [28].

Our findings regarding health-related quality of life are in line with previously published data. Koivusalo et al. assessed QoL using *SF-36* questionnaire in adults with congenital abdominal wall defects and found no significant differences compared with the general population [29]. Likewise, Kapapa et al. reported comparable quality of life outcomes in children with gastroschisis and omphalocele using the KINDL questionnaire, with no relevant deviations from healthy peers [25].

Our results indicate important implications for both initial care and long-term follow-up of patients with abdominal wall defects. Parents may overestimate the impact of scars, while children often demonstrate remarkable resilience. Nevertheless, complaints such as itching, hypersensitivity, and pain should be addressed, as caregivers may underestimate their effect on daily life. Prospective studies are needed to validate these observations.

This study is limited by its single-center design and relatively small cohort. With a minimum age of 11 years at query, we opted for a reasonable age to assess scars and well-being, even if age might influence results within an enlarged cohort. We aspire to publish age-adjusted research on this topic in large-population-studies. Subanalyses adjusted for age, dimensions of abdominal wall defects or further abdominal surgeries revealed merely significant results, although they were expected. We waived multivariate analyses due to the small sample size and look forward to enlarged population studies to confirm these hypotheses. However, our study benefits from a consistent surgical approach and the use of validated questionnaires, including the *POSAS* for detailed pain assessment. While *PSAQ* provided useful information, it has not yet been validated for comparative purposes. Future research should also consider evolving surgical techniques, such as suture-less closure, as potential variables in long-term outcomes.

## Conclusion

In our collective, there is a clear difference in the self- and third-party assessment of scar quality between patients and parents. Treating physicians should be aware that parents may overestimate the negative appearance of scars while underestimating their child’s pain and increased sensitivity. Primarily larger and multi-center studies are needed to confirm the presented data.

## Data Availability

No datasets were generated or analysed during the current study.
